# Dynamic light caused less photosynthetic suppression, rather than more, under nitrogen deficit conditions than under sufficient nitrogen supply conditions in soybean

**DOI:** 10.1186/s12870-020-02516-y

**Published:** 2020-07-17

**Authors:** Yu-Ting Li, Ying Li, Yue-Nan Li, Ying Liang, Qiang Sun, Geng Li, Peng Liu, Zi-Shan Zhang, Hui-Yuan Gao

**Affiliations:** 1 State Key Lab of Crop Biology, Tai’an, Shandong Province China; 2 College of Agronomy, Shandong Agricultural University, Tai’an, Shandong Province China; 3 College of Life Sciences, Shandong Agricultural University, Tai’an, Shandong Province China; 4 Tai’an Testing Center For Food And Drug Control, Tai’an, Shandong Province China

**Keywords:** Dynamic light, Low nitrogen, Photosynthesis, Soybean

## Abstract

**Background:**

Plants are always exposed to dynamic light. The photosynthetic light use efficiency of leaves is lower in dynamic light than in uniform irradiance. Research on the influence of environmental factors on dynamic photosynthesis is very limited. Nitrogen is critical for plants, especially for photosynthesis. Low nitrogen (LN) decreases ribulose-1,5-bisphosphate carboxylase/oxygenase (Rubisco) and thus limits photosynthesis. The decrease in Rubisco also delays photosynthetic induction in LN leaves; therefore, we hypothesized that the difference of photosynthetic CO_2_ fixation between uniform and dynamic light will be greater in LN leaves compared to leaves with sufficient nitrogen supply.

**Results:**

To test this hypothesis, soybean plants were grown under low or high nitrogen (HN), and the photosynthetic gas exchange, enzyme activity and protein amount in leaves were measured under uniform and dynamic light. Unexpectedly, dynamic light caused less photosynthetic suppression, rather than more, in LN leaves than in HN leaves. The underlying mechanism was also clarified. Short low-light (LL) intervals did not affect Rubisco activity but clearly deactivated fructose-1,6-bisphosphatase (FBPase) and sedoheptulose-1,7-bisphosphatase (SBPase), indicating that photosynthetic induction after a LL interval depends on the reactivation of FBPase and SBPase rather than Rubisco. In LN leaves, the amount of Rubisco decreased more than FBPase and SBPase, so FBPase and SBPase were present in relative excess. A lower fraction of FBPase and SBPase needs to be activated in LN leaves for photosynthesis recovery during the high-light phase of dynamic light. Therefore, photosynthetic recovery is faster in LN leaves than in HN leaves, which relieves the photosynthetic suppression caused by dynamic light in LN leaves.

**Conclusions:**

Contrary to our expectations, dynamic light caused less photosynthetic suppression, rather than more, in LN leaves than in HN leaves of soybean. This is the first report of a stress condition alleviating the photosynthetic suppression caused by dynamic light.

## Background

Plants growing under natural or semi-natural conditions are exposed to a dynamic light environment [1–3]. The light intensity on the surface of leaves varies over time scales ranging from seconds to hours. This variation is due to the movement of the sun, changes in cloud cover and shade from neighbouring plants [4–6]. Therefore, for a more comprehensive understanding of the response of photosynthesis to the environment, it is essential to characterize photosynthesis under dynamic light conditions [2, 6].

Compared with uniform irradiance, dynamic light decreases the light use efficiency of leaves [1–4, 7] due to (1) the delay in photosynthetic recovery after switching from low light (LL) to high light (H) [6, 8, 9]; (2) the postillumination photorespiratory CO_2_ burst [6, 10, 11]; and (3) the relaxation lag of nonphotochemical quenching (NPQ) after switching from HL to LL [12, 13]. To effectively utilize the energy in dynamic light, plants need to activate photosynthesis quickly when HL conditions occur. Fast photosynthetic induction requires the rapid activation of Ribulose-1,5-bisphosphate carboxylase / oxygenase (Rubisco) and other Calvin cycle enzymes as well as the rapid opening of stomata [2, 8, 14]. The catalytic efficiency of Rubisco is relatively low, and the photosynthesis flux control value of Rubisco is much higher than the control values of other enzymes in the Calvin cycle [15]. In addition, the activation rate of Rubisco is relatively slow; therefore, Rubisco is considered the limiting factor of photosynthetic induction under most conditions [2, 16]. It has been reported that the speed of the in vivo activation of Rubisco is responsible for different photosynthetic induction rates among genotypes of soybean [17]. The activation of Rubisco is dependent on an auxiliary enzyme, Rubisco activase (RCA) [18, 19]. RCA maintains Rubisco activity by facilitating the release of inhibitory sugar molecules that impede carbamylation or catalysis [20, 21]. Recent studies have reported that the amount of RCA is closely related to the photosynthetic induction rate [22], and improved RCA proteins clearly accelerate photosynthetic induction [23].

Nitrogen is one of the most important inorganic nutrients in plants. Nitrogen is crucial for plant growth, yield and especially photosynthesis, as more than 50% of total leaf nitrogen is allocated to the photosynthetic apparatus [24, 25]. Low nitrogen (LN) supply will decrease the amount of photosynthetic pigments, thylakoid membrane proteins and Calvin cycle-related enzymes, thereby reducing photosynthetic CO_2_ fixation in leaves [26]. Rubisco, the rate-limiting enzyme of photosynthetic CO_2_ fixation in C3 leaves under normal atmospheric CO_2_ conditions, is one of the proteins most sensitive to nitrogen supply levels because it is the most abundant enzyme in plant leaves and contains a large amount of nitrogen (approximately 20% of leaf nitrogen) [27].

To compensate for the decrease in Rubisco content, Rubisco activation increases in leaves with LN supply [28–32]. During photosynthetic induction, a higher Rubisco activation state will take longer to achieve in LN supply leaves. Therefore, LN supply may decelerate photosynthetic induction and thus decrease light use efficiency under dynamic light. Therefore, it was hypothesized that the difference of photosynthetic CO_2_ fixation between uniform and dynamic light will be greater in LN leaves compared to leaves with sufficient nitrogen supply.

To verify the above hypothesis, soybean seedlings were grown under two nitrogen supply levels. Then, the photosynthetic gas exchange, activity and amount of Calvin cycle enzyme proteins in these leaves were analysed under dynamic and uniform irradiance. Although the rhizobia that are symbiotic with soybeans can fix nitrogen, soybeans in the field still rely on the application of large amounts of fertilizer, especially during the rotation of soybeans with other crops. Nitrogen deficiency often occurs in soybean cultivation.

## Results

### Photosynthetic gas exchange under steady-state light conditions

**Fig. 1:**
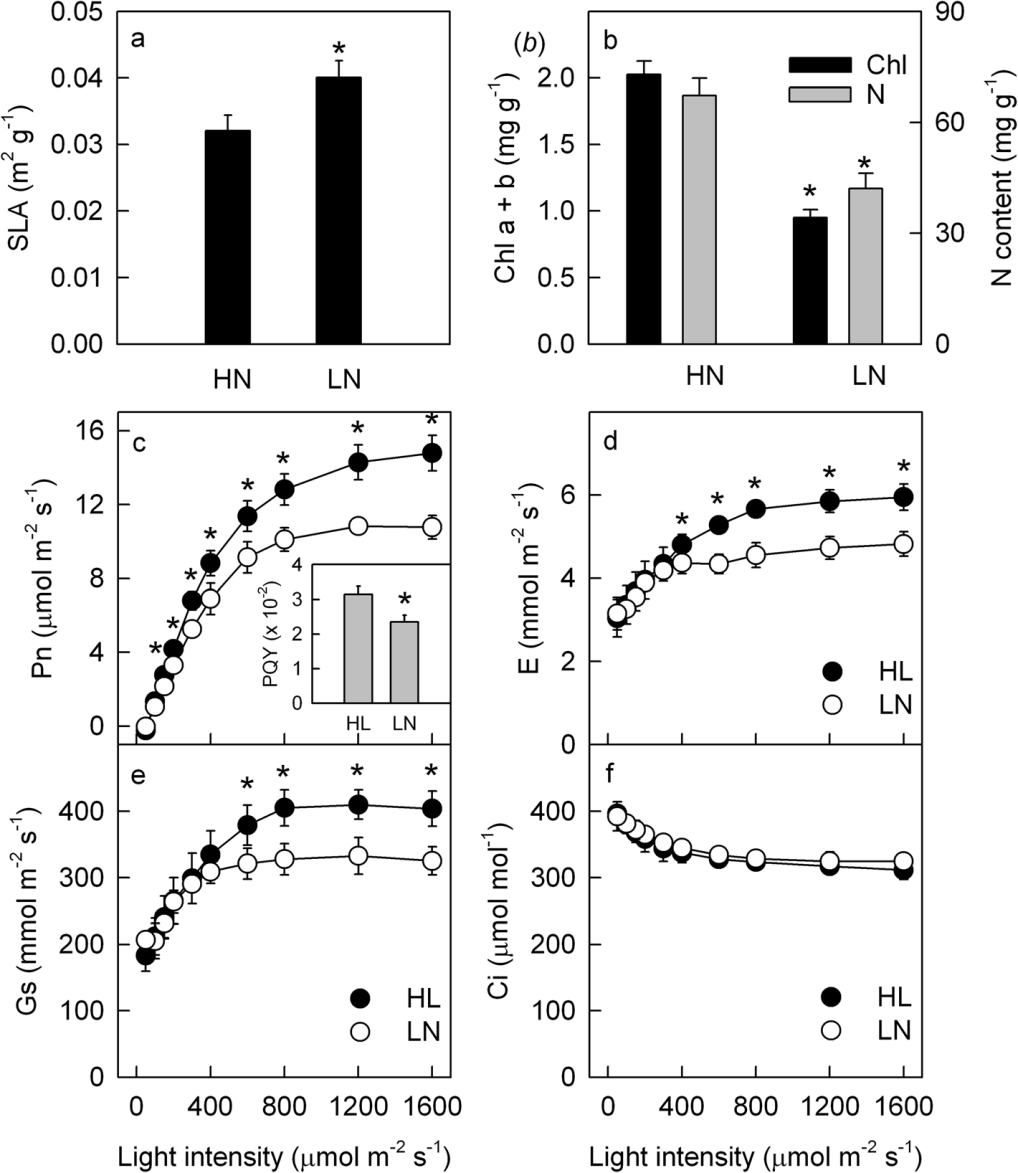
Substance content and steady-state photosynthetic gas exchange. The specific leaf area (SLA; **a**), total chlorophyll (Chl) and nitrogen (N) contents (**b**), light intensity response curve of the net photosynthetic rate (Pn; **c**), transpiration rate (E; **d**), stomatal conductance (Gs; **e**) and intercellular CO_2_ concentration (Ci; **f**) as well as the photosynthetic quantum yield (PQY; plot c insert) in the leaves of high nitrogen (HN; filled)- and low nitrogen (LN; closed)-supplied plants. Means ± SD, *n* = 6. The asterisks indicate significant differences at *P* < 0.05 between HN and LN leaves (T-test)

Low N (LN) supply significantly decreased the contents of nitrogen and chlorophyll and increased the specific leaf area (SLA; Fig. 1a, b). In LN leaves, the steady-state Pn was lower than that in HN leaves under 150–1600 μmol m^− 2^ s^− 1^ illumination (Fig. 1c). The lower PQY [33], which was estimated as the initial slope of the linear relationship between the Pn and PFD in LL leaves (Fig. 1c), indicated that the efficiency of light energy use under limited light conditions was restrained by LN supply. The Pn under saturating light (1200–1600 μmol m^− 2^ s^− 1^) was lower in the LN leaves than in the HN leaves (Fig. 1c), which indicated that the photosynthetic capacity was lower in the LN leaves. Furthermore, compared with the HN leaves, the LN leaves exhibited low stomatal conductance (Gs) and a low transpiration rate (E) but similar intercellular CO_2_ concentration (Ci; Fig. 1d-f), indicating that the low Pn in the LN leaves was caused by nonstomatal limitation.

### Photosynthetic gas exchange under changing light conditions

**Fig. 2:**
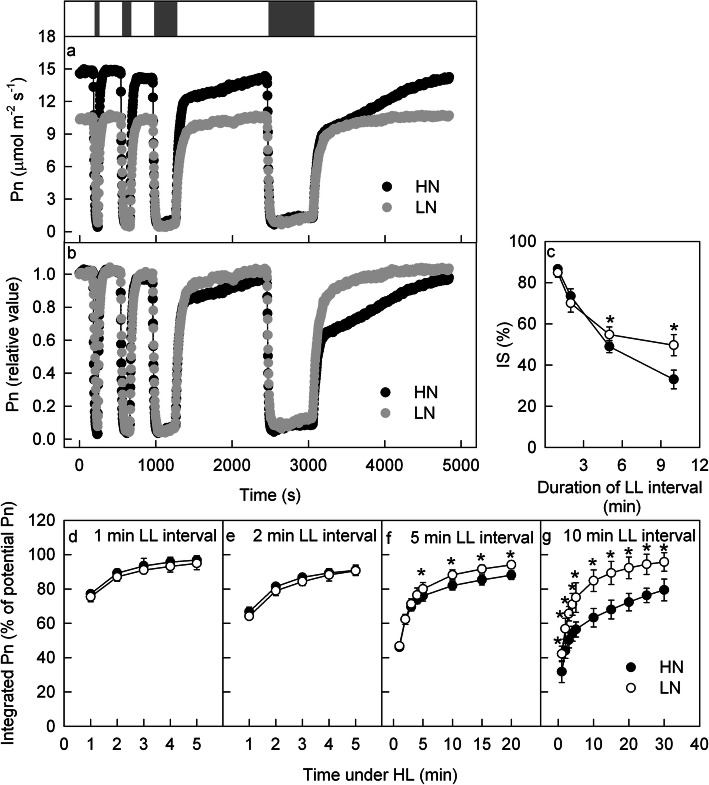
Photosynthetic gas exchange under changing light conditions. The time course of the net photosynthetic rate (Pn; **a**, **b**) under changing light in the leaves of high nitrogen (HN; black) and low nitrogen (LN; grey) supply plants. The bar above the plot (**a**) shows the high (1600 μmol m^− 2^ s^− 1^; HL; white bar) and LL (100 μmol m^− 2^ s^− 1^; LL; grey bar) periods. The leaves were adapted under HL for 20–40 min until the Pn stabilized, after which the leaves were exposed to changing light. The grey bars, from left to right, represent 60, 120, 300, and 600 s of LL. The original Pn is shown in plot (**a**). In plot (**b**), the Pn under steady HL was taken as 100%, and the Pn under changing light conditions was calculated as a percentage of the Pn under steady HL. (**c**) The induction state of Pn (IS%) after LL intervals of different durations. (**d**-**g**) The integrated Pn during HL following 60 (**d**), 120 (**e**), 300 (f) or 600 s (**g**) LL intervals. Means ± SD, n = 6. The asterisks indicate significant differences at P < 0.05 between HN and LN leaves (T-test)

To clarify the effects of N supply on dynamic photosynthesis, HL-adapted (1600 μmol m^− 2^ s^− 1^) leaves were exposed to intervals of LL (100 μmol m^− 2^ s^− 1^) for various durations; afterward, the LL was switched to HL (as shown in the bar above Fig. 2a), and the photosynthetic gas exchange was synchronously recorded. When the light intensity decreased, the Pn decreased quickly; however, when the light intensity increased, the Pn could not recover immediately but increased gradually (Fig. 2a, b). After a short low-light interval (60 or 120 s), the Pn showed similar recovery under HL conditions in the HN and LN leaves. However, when the low-light intervals were extended to 300 or 600 s, the subsequent recovery of Pn under HL in the LN leaves was faster than that in the HL leaves (Fig. 2a, b). The Pn after 30 s of HL exposure was used to indicate the IS% after low-light intervals [8, 9]. The IS% gradually decreased with extended duration of the low-light intervals; the IS% after a 300 or 600 s low-light interval was significantly lower in the HN leaves than in the LN leaves (Fig. 2c).

To quantify the effects of dynamic light on photosynthetic carbon fixation, the integrated Pn during HL after various durations of low-light intervals was calculated (Fig. 2d-g). The low-light intervals caused significant losses of carbon fixation during the subsequent high-light period; this loss was aggravated by longer low-light intervals, and this loss was more severe at the beginning of the HL conditions (Fig. 2d-g). The loss of photosynthetic carbon fixation after a 60 or 120 s low-light interval was similar between the LN and HN leaves, but the loss after a 300 or 600 s low-light interval was more severe in the HN leaves than in the LN leaves (Fig. 2d-g).

In agreement with the earlier reports [34], under changing light conditions, the Gs and E exhibited a similar trend to that of Pn, but the Gs and E changed more slightly and slowly than did the Pn (Additional file 1). We also observed that the Gs and E decreased more sharply in the HN leaves than in the LN leaves. The change in Ci was similar in the HN and the LN leaves under changing light conditions (Additional file 1), which suggested that the different dynamic Pns between the HN and LN leaves under changing light conditions were due to nonstomatal limitations.

PSII photoinhibition due to HL exposure is also a potential factor that affects CO_2_ fixation. Hence, the maximum quantum yield of PSII (Fv/Fm) [35, 36] and the amounts of D1 protein, a core subunit of the PSII reaction centre in leaves [37–39], were measured before and after dynamic photosynthetic gas exchange measurement to reflect PSII photoinhibition. The dynamic photosynthetic gas exchange measurement caused a slight but significant decrease in Fv/Fm; however, the Fv/Fm was similar in LN and HN leaves (Additional file 2). In addition, the amounts of D1 protein did not change after photosynthetic gas exchange measurement (Additional file 2). The similar Fv/Fm between HN and LN leaves and stable D1 protein after dynamic photosynthetic gas exchange measurement indicated that the different dynamic Pns between the HN and LN leaves were independent of PSII photoinhibition and D1 protein degradation.

Not only the N content but also the osmotic potential was different between the HN and LN nutrient solutions. To analyse the effect of osmotic potential on photosynthetic gas exchange, we added polyethylene glycol (PEG) to the LN nutrient solution and ensured that the osmotic potential in the LN + PEG nutrient solution was similar to the osmotic potential in the HN nutrient solution. The LN plant was moved to the LN + PEG nutrient solution, and the photosynthetic gas exchange in leaves was measured before and 24 h after moving to the LN + PEG nutrient solution. The photosynthetic gas exchange was not affected by the added PEG (Additional file 3). Therefore, the lower osmotic potential in the HN nutrient solution did not influence the photosynthetic gas exchange.

### Photosynthetic gas exchange under fluctuating light conditions

**Fig. 3:**
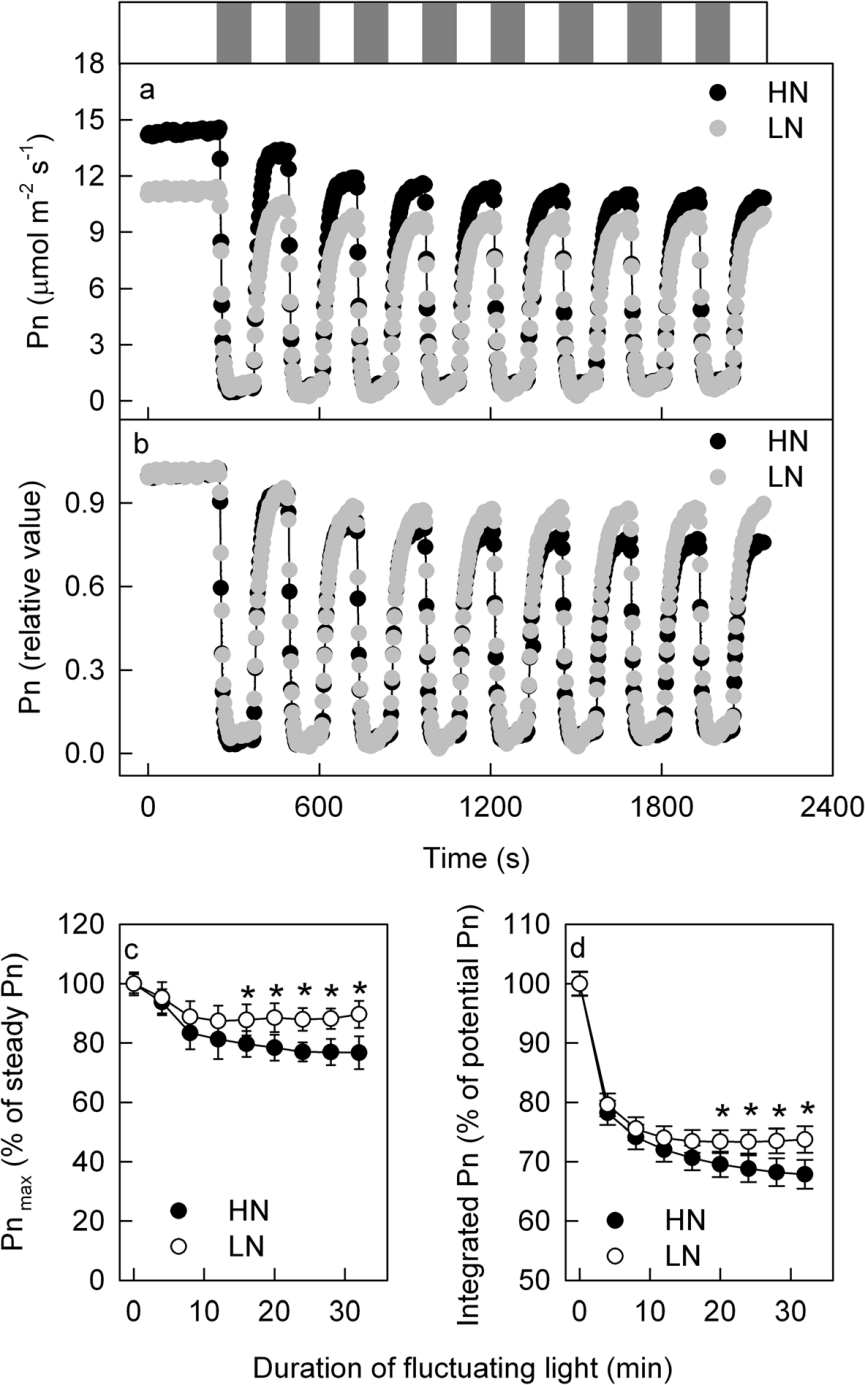
Photosynthetic gas exchange under fluctuating light conditions. The time course of the net photosynthetic rate (Pn; **a**, **b**) under fluctuating light in the leaves of high nitrogen (HN; black) and low nitrogen (LN; grey) supply plants. The bar above the plot (**a**) shows the high (1600 μmol m^− 2^ s^− 1^; HL; white bar) and LL (100 μmol m^− 2^ s^− 1^; LL; grey bar) periods. The leaves were adapted under HL (1600 μmol m^− 2^ s^− 1^) for 20–40 min until the Pn stabilized, after which the leaves were exposed to fluctuating light such that the light intensity alternated between high (1600 μmol m^− 2^ s^− 1^) and low (100 μmol m^− 2^ s^− 1^) conditions every 120 s. The original Pn is shown in plot (**a**). In plot (**b**), the Pn under steady HL was taken as 100%, and the Pn under changing light conditions was calculated as a percentage of the Pn under steady HL. (**b**) The maximum Pn during the HL period (Pn_max_) in HN- and LN-supplied plants; the Pn under steady HL was taken as 100%, and the Pn_max_ was calculated as a percentage of the Pn under steady HL. (**c**) The integrated Pn during fluctuating light in HN- and LN-supplied plants. Means ± SD, n = 6. The asterisks indicate significant differences at P < 0.05 between HN and LN leaves (T-test)

The above results showed that the responses of photosynthetic gas exchange in LN and HN leaves to one short low-light interval (60 or 120 s) were similar, but their responses under recurring short low-light intervals, which are common in field conditions, were unknown. To clarify this uncertainty, the leaves were exposed to light regimes that fluctuated between 100 and 1600 μmol m^− 2^ s^− 1^ every 120 s for a total of 32 min (Fig. 3a, b). It was observed that during the 120 s high-light period after a 120 s low-light interval, the Pn could not recover to the level under steady-state HL conditions, so the maximum Pn under the HL period (Pn_max_) gradually decreased with additional fluctuating light durations (Fig. 3c). Due to the delayed induction of Pn and the decrease in Pn_max_, the integrated Pn was lower under fluctuating light than under steady-state light, and this loss of integrated Pn was aggravated by increased durations of fluctuating light (Fig. 3d). These decreases in Pn_max_ and the integrated Pn under fluctuating light were more obvious in the HN leaves than in the LN leaves.

Under fluctuating light conditions, Gs and E decreased gradually, and the decreases in Gs and E were more severe in the HN leaves than in the LN leaves (Additional file 4). Moreover, the change in Ci was similar in the HN and LN leaves under fluctuating light (Additional file 4), which indicated that the different dynamic Pns between the HN and LN leaves under fluctuating light were due to nonstomatal limitations. The Fv/Fm slightly but significantly decreased after photosynthetic gas exchange measurement under fluctuating light conditions, but the decrease in Fv/Fm was similar in LN and HN leaves, which indicated that the different dynamic Pns under fluctuating light conditions between the HN and LN leaves was independent of PSII photoinhibition.

### RuBP carboxylation and regeneration capacity

**Fig. 4:**
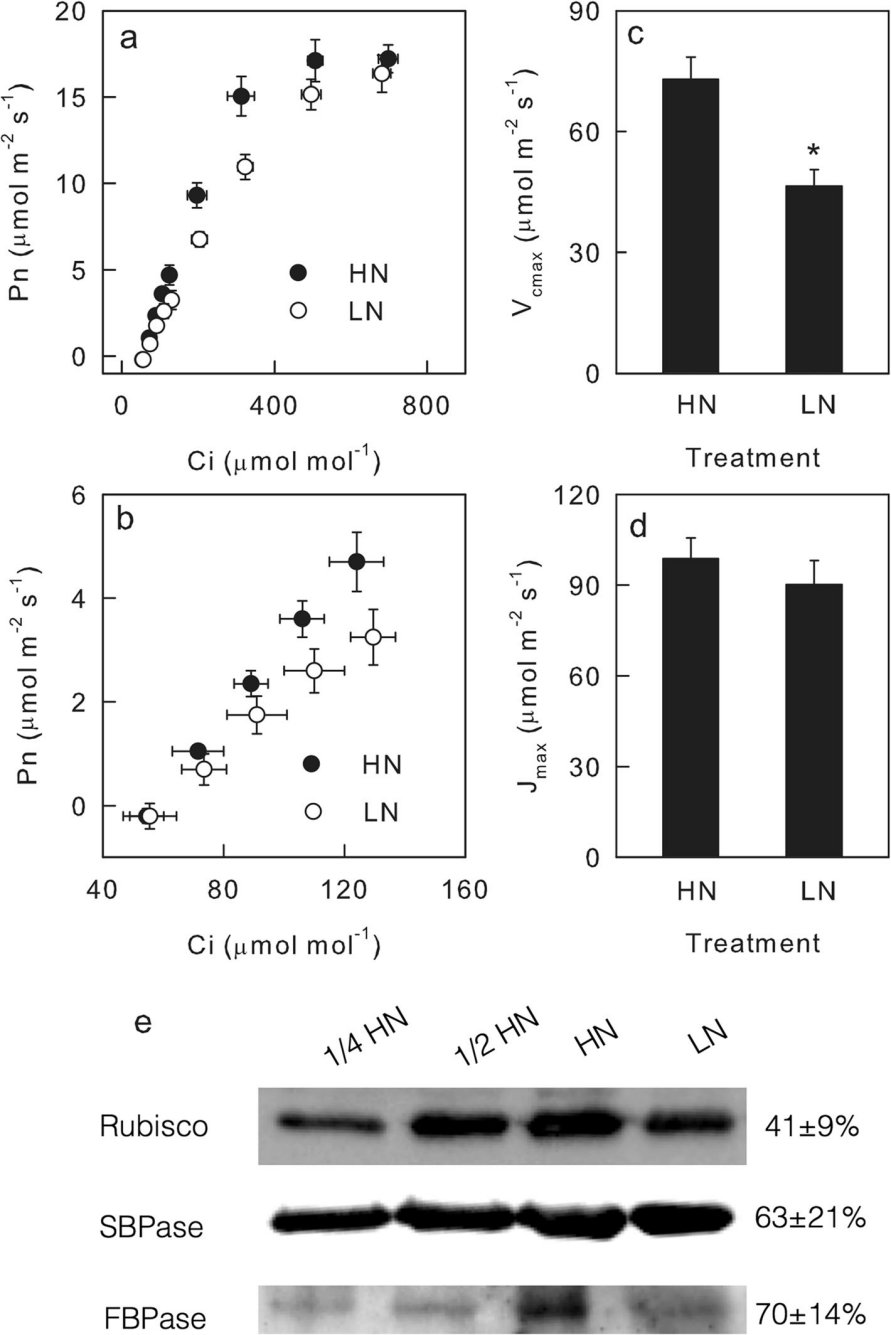
RuBP carboxylation and regeneration capacity. The intercellular CO_2_ concentration (Ci) response curve of the net photosynthetic rate (Pn; **a**, **b**); the maximum rates of RuBP-carboxylation (V_cmax_; **c**); the maximum rates of RuBP regeneration (J_max_; **d**); and the amounts of Rubisco, SBPase and FBPase (**e**) in the leaves of high nitrogen (HN; filled)- and low nitrogen (LN; closed)-supplied plants. In plot e, 1/2 and 1/4 indicate the quantity of protein sample loaded, and the number to the right of the bands indicates the protein content in LN leaves as a percentage of that in HN leaves. The original, full-length gel and blot were listed in Additional file 5. Means ± SD, n = 6 (gas exchange) or 3 (immunoblot). The asterisks indicate significant differences at P < 0.05 between HN and LN leaves (T-test)

The above results suggested that the difference in dynamic photosynthesis between LN and HN leaves was mostly caused by nonstomatal limitations. In addition to stomatal limitations, photosynthetic carbon fixation could be limited by biochemical factors, which include RuBP carboxylation and regeneration [40–42]. Therefore, the Pn/Ci response curve was measured under saturating light (1600 μmol m^− 2^ s^− 1^) to quantify the RuBP carboxylation and regeneration capacity of leaves. The Pn was higher in the HN leaves than in the LN leaves when the Ci was lower than 600 μmol mol^− 1^ (Fig. 4a, b), but the difference in Pn between the HN and LN leaves diminished under saturated Ci (Fig. 4a). The V_cmax_ was significantly lower in the LN leaves than in the HN leaves; however, the J_max_ values in LN and HN leaves were similar (Fig. 4c, d).

The immunoblot analysis showed that the content of Rubisco, the key enzyme of RuBP carboxylation, decreased by over 50% in the LN leaves compared with the HN leaves; however, the contents of SBPase and FBPase, the key enzymes in RuBP regeneration, decreased by less than 50% in the LN leaves compared with the HN leaves (Fig. 4e). These results supported the gas exchange data in that a low N supply decreased the RuBP regeneration capacity more severely than the RuBP carboxylation capacity.

### The enzyme activity under steady and dynamic conditions

**Fig. 5:**
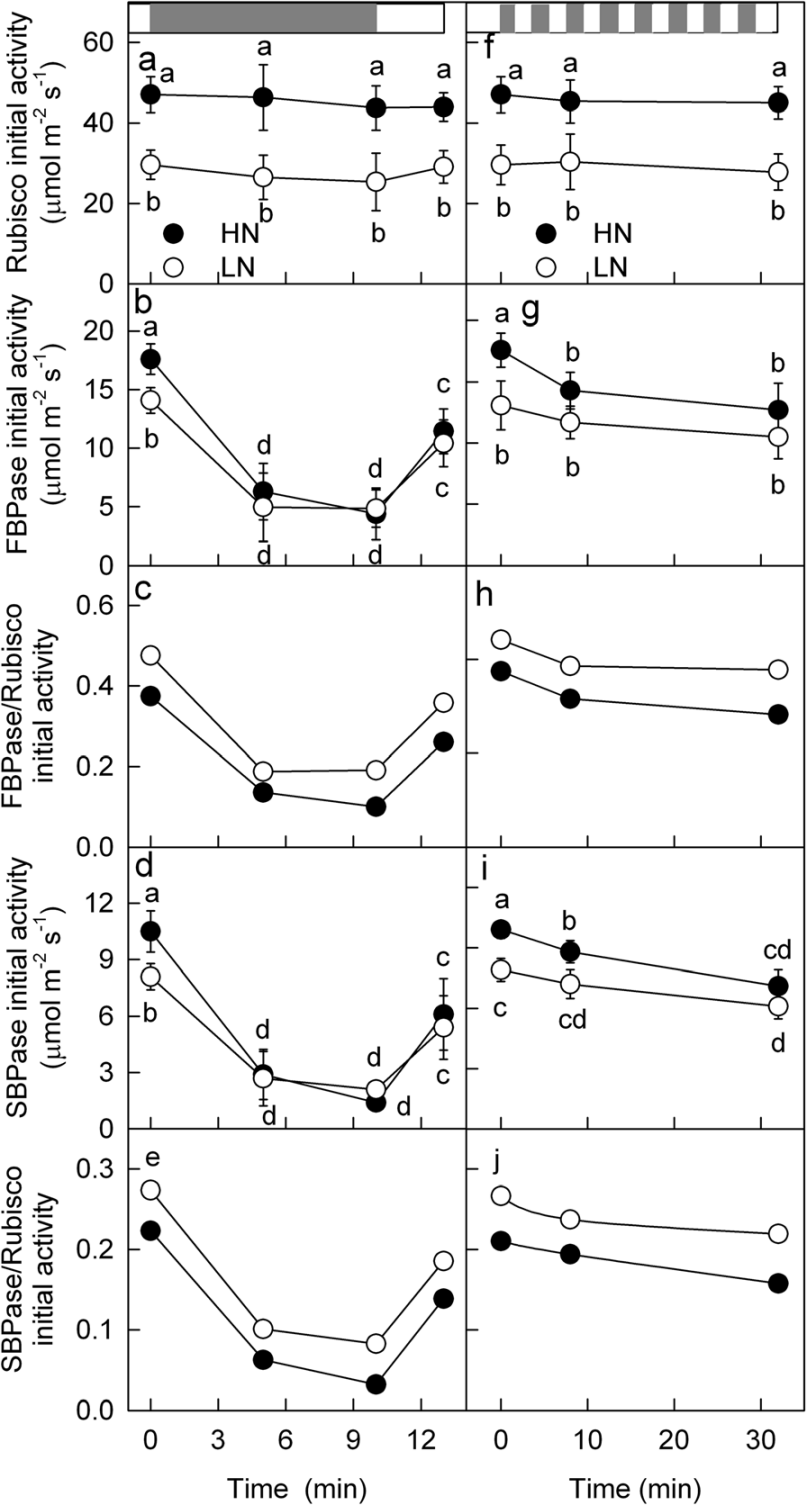
Enzyme activity under steady and dynamic conditions. The activity of Rubisco (**a**, **f**), FBPase (**b**, **g**) and SBPase (**d**, **i**) as well as the ratios of activity between FBPase and Rubisco (**c**, **h**) and between SBPase and Rubisco (**e**, **j**) in the leaves of high nitrogen (HN; filled)- and low nitrogen (LN; closed)-supplied plants under dynamic light. The bar above the plot (**a**) shows the high (1600 μmol m^− 2^ s^− 1^; HL; white bar) and LL (100 μmol m^− 2^ s^− 1^; HL; grey bar) periods. The leaves under changing light (**a**-**e**) were adapted under HL for 20–40 min, and the leaves were then exposed to LL for 600 s, after which the light was changed to HL for 180 s. The leaves under fluctuating light (**f**-**j**) were adapted under HL for 20–40 min, after which the leaves were exposed to fluctuating light such that the light intensity alternated between high (1600 μmol m^− 2^ s^− 1^) and low (100 μmol m^− 2^ s^− 1^) every 120 s for 32 min. Means ± SD, n = 6. Different letters indicate significant differences at P < 0.05 between different treatments (T-test)

The amount of protein reflects the potential capacity of RuBP carboxylation and regeneration in LN and HN leaves but does not reflect the actual activity of related enzymes, especially under changing light conditions. To further clarify the mechanism underlying the different dynamic photosynthesis processes between the LN and HN leaves, the activity levels of Rubisco, FBPase and SBPase were measured under steady and changing light conditions. Under steady HL, the activity levels of Rubisco, FBPase and SBPase were all lower in the LN leaves than in the HN leaves, but the difference in Rubisco activity between the LN and HN leaves was more pronounced than the differences in FBPase and SBPase activity; the ratios of FBPase/Rubisco and SBPase/Rubisco activities were higher in the LN leaves than in the HN leaves (Fig. 5). After a 600 s low-light interval, the activity of Rubisco remained almost steady, but the FBPase and SBPase activity decreased to a very low level in both the LN and HN leaves (Fig. 5). Next, when the leaves were exposed again to HL, the activity of FBPase and SBPase in the LN and HN leaves increased at the same rate. Moreover, after the leaves were exposed to fluctuating light for 32 min, the Rubisco activity did not change in either the LN or HN leaves, but FBPase was deactivated in HN leaves more than in LN leaves, and SBPase was deactivated by 20.2% in HN leaves but only 14.1% in LN leaves. In addition, under dynamic light conditions, the ratios of FBPase/Rubisco and SBPase/Rubisco activity were always higher in the LN leaves than in the HN leaves.

## Discussion

### RuBP regeneration-related enzymes limit photosynthetic induction under dynamic light

Dynamic light consists of HL sunflecks and LL intervals between sunflecks. The fast photosynthetic activation during HL sunflecks, as well as the maintenance of the photosynthetic activation state during the LL interval, could contribute to the efficient utilization of dynamic light. Therefore, both enzymes that are activated slowly under HL sunflecks and enzymes whose activity decreases rapidly during LL intervals are potential limiting factors for optimizing photosynthesis under dynamic light conditions.

One 600 s LL interval or recurring shorter (120 s) LL intervals interfered only slightly with Rubisco activity but markedly reduced the activity of FBPase and SBPase (Fig. 5), which is consistent with the faster reduction and oxidation of FBPase proteins under light and dark conditions, respectively [43]. Next, when HL suddenly appears, Rubisco is still highly active, but SBPase and FBPase need to be reactivated to match the activity of Rubisco. Therefore, photosynthetic induction after an LL interval is limited mainly by the reactivation of SBPase and FBPase.

SBPase and FBPase were almost completely deactivated after a 10 min LL interval (Fig. 5), so a longer LL interval will not continue deactivating SBPase and FBPase. However, the activity of Rubisco will continue to decrease if the duration of the LL interval is extended. Therefore, we presume that the limitation of Rubisco activation state to photosynthetic induction will increase with the extension of LL interval.

In addition to SBPase and FBPase, which were measured in this experiment, GAPDH and PRK are also light-activated enzymes and potential rate-limiting enzymes in RuBP regeneration [15], though their photosynthetic flux control values are lower than those of SBPase and FBPase [15]. Both in vitro and in vivo experiments have shown that the GAPDH and PRK enzymes were activated very quickly but inactivated slowly [44–46], even under only 300 μmol m^− 2^ s^− 1^ [41]. However, opposite results were also reported [47]. Moreover, some nonregulated enzymes that catalyse reversible reactions, such as aldolase and transketolase, exert significant control over carbon flux under steady-state light [15, 48–50]. Therefore, the roles of other Calvin cycle-related enzymes in photosynthetic induction under dynamic light require further study.

Recent studies have reported that redundant NPQ can decrease photosynthetic electron transport and thus restrict CO_2_ fixation under dynamic light conditions [12, 51]. However, we think the difference in dynamic photosynthesis between LN and HN soybean leaves was not contributed by the NPQ itself because electron transport is the limiting factor of CO_2_ fixation only under low light and not under high light; thus, NPQ influenced electron transport and therefore changed CO_2_ fixation after the conversion from high to low light rather than after the conversion from low to high light [12, 51]. The different CO_2_ fixation behaviours between LN and HN leaves were observed only after the conversion from low to high light (Figs. 2, 3), so this difference was not caused by NPQ.

### The dynamic light-induced photosynthetic suppression is less, rather than more, in LN leaves than in HN soybean leaves

Like steady-state photosynthesis, dynamic photosynthesis can also be influenced by environmental factors. The relationship between steady-state photosynthesis and the environment has been extensively researched; however, there are only a limited number of studies related to the influence of environmental factors on dynamic photosynthesis [2]. Generally, stress conditions will increase the photosynthetic suppression caused by dynamic light, and favourable conditions will alleviate the effect. It was reported that drought [9], nitrogen deficiency [8] and heat stress [52] all increased the photosynthetic suppression caused by dynamic light. In addition, the dynamic light-induced photosynthetic suppression was alleviated by a high CO_2_ atmosphere [10, 53–56]. However, our results showed that dynamic light-induced photosynthetic suppression was alleviated rather than exacerbated by LN stress. As far as we know, this research is the first to report that a stress condition alleviates the photosynthetic suppression caused by dynamic light.

This result was outside our expectations. According to traditional viewpoints [9], the activation state of Rubisco will increase to compensate for the decreased amount of Rubisco in LL supply leaves; a higher Rubisco activation state will take longer to achieve, which will delay photosynthetic induction and therefore increase the photosynthetic suppression caused by dynamic light.

However, this study indicated that photosynthetic induction after the LL interval is limited mainly by the reactivation of SBPase and FBPase rather than by Rubisco (see above). Therefore, although the content of Rubisco decreased significantly in LN supply leaves (Figs. 4, 5), this decrease did not delay photosynthetic induction after LL intervals. In addition, because the amount of Rubisco decreased more obviously than the amounts of SBPase and FBPase (Fig. 4), SBPase and FBPase were present in relative excess in LN leaves (Fig. 5). Therefore, a lower fraction of SBPase and FBPase was required for activation (Fig. 5), and thus, a shorter time was needed in the LN leaves to match the activity of Rubisco during photosynthetic induction after LL intervals. Consequently, photosynthetic induction was faster in LN supply leaves than in HN supply leaves, alleviating the dynamic light-induced photosynthetic suppression.

## Conclusions

This study suggests that the reactivation of RuBP regeneration-related enzymes (SBPase and FBPase), rather than RuBP carboxylation enzyme (Rubisco), limits photosynthetic induction and light use efficiency under dynamic light. Excess FBPase and SBPase relative to Rubisco in LN soybean leaves accelerated photosynthetic induction under dynamic light. Therefore, contrary to our expectations, dynamic light caused less photosynthetic suppression, rather than more, in LN leaves than in HN soybean leaves.

## Methods

### Plant materials

Soybean (*Glycine max* cv. Qihuang34; purchased from Crop Research Institute, Shandong Academy of Agricultural Sciences) undertook the formal identification. The seeds are still sold commercially, but it is not clear whether the voucher specimen has been deposited in a publicly available herbarium. Plants were grown in pots (20 cm in diameter, 25 cm in height) filled with vermiculite. Nutrient solutions with high (10 mM nitrate) or low (1 mM nitrate) nitrogen were used to irrigate plants every 2 days. The nutrient solution contained 1 or 10 mM N, 10 mM P, 25 mM K, 2 mM Mg, 1 mM Ca, 2 mM S, 0.1 mM Fe, 180 μM B, 25 μM Mn, 3 μM Zn, 1.3 μM Cu, and 0.5 μM Mo. Not only nitrogen deficiency but also excess nitrogen is harmful to plants, including the photosynthetic mechanism. The 10 mM nitrate used as “high nitrogen treatment” in this study has been referred to in previous reports in soybean [57, 58] and other species [59–62] as supplying sufficient but not excess nitrogen to plants.

The different levels of nitrogen supply also affect the osmotic potential of the nutrient solutions, but this mild increase in the osmotic potential of high nitrogen (HN) nutrient solutions can be neglected. Soil was not used during growth, and nodulation was not observed during the experiment. The plants were placed in a greenhouse; the maximum temperature and light intensity were approximately 32 °C and 1000 μmol m^− 2^ s^− 1^ during the day. The humidity in the greenhouse was 50–70%. The youngest fully developed leaves of 4- to 5-week-old plants were used for the experiments.

### SLA, nitrogen and chlorophyll content

The specific leaf area (SLA) was calculated by dividing the leaf area by the leaf dry mass. The leaf area was measured using an LI-3000C Area Meter (Li-Cor, USA), and then the leaf material was dried at 70 °C. The leaf chlorophyll was extracted with 80% acetone from frozen leaves, and the extracts were analysed with a UV-2550 spectrophotometer (Shimadzu, Japan) in accordance with the methods of Porra et al. [63]. The total nitrogen content was measured with a K9860 automatic Kjeldahl apparatus (Hanon, China).

### Gas exchange measurements

The photosynthetic gas exchange parameters, photosynthetic rate (Pn), transpiration rate (E) and substomatal CO_2_ concentration (Ci) were measured using a CIRAS-3 portable photosynthesis system (PP Systems, USA). The light intensity, light quality (95% red and 5% blue light), CO_2_ partial pressure (Cr), relative humidity (60%) and leaf temperature (30 °C) were controlled by an automatic control device in the CIRAS-3 photosynthesis system. Before the photon flux density (PFD) and CO_2_ response curve measurements, the leaves were adapted to HL (1600 μmol m^− 2^ s^− 1^) and normal CO_2_ (400 μmol mol^− 1^) conditions, and they were then adapted to each light intensity and CO_2_ concentration for at least 120 s during the response curve measurements. The light intensities were altered in the following order during the measurement of the PFD response curve: 1600, 1200, 800, 600, 400, 300, 200, 150, 100, and 50 μmol m^− 2^ s^− 1^. The Cr was altered in the following order during the measurement of the CO_2_ response curve: 400, 300, 200, 175, 150, 125, 100, 400, 600, and 800 μmol mol^− 1^. The photosynthetic quantum yield (PQY) was estimated as the slope of the linear relationship between Pn and light intensity under LL (50–200 μmol m^− 2^ s^− 1^) [33]. Measurements for the Pn/Ci response curves were made starting at 400 μmol mol^− 1^ ambient CO_2_ and then decreased stepwise to 50 μmol mol^− 1^; afterward, the concentration was returned to 400 μmol mol^− 1^ and maintained for at least 15 min until the Pn was restored to the initial value to reactivate Rubisco, and then, the ambient CO_2_ concentration was gradually increased from 400 to 900 μmol mol^− 1^. The CO_2_ assimilation rate is limited either by RuBP carboxylation or by RuBP regeneration according to the C3 photosynthesis model [40]. The maximum rates of RuBP-carboxylation (V_cmax_) and RuBP-regeneration (J_max_) were calculated by fitting the Pn/Ci response curves [64, 65]. A typical value for mitochondrial respiration rates in the light (Rd), the Michaelis constant of Rubisco for carbon dioxide and the CO_2_ compensation point in the absence of respiration (1.35 μmol m^− 2^ s^− 1^, 651 μmol mol^− 1^, and 55.2 μmol mol^− 1^, respectively) [65] were used to solve the values of V_cmax_ and J_max_ in this study.

To measure dynamic gas exchange under changing light conditions, the leaves were light adapted under 1600 μmol m^− 2^ s^− 1^ light and 400 μmol mol^− 1^ CO_2_ in the chamber of the CIRAS-3. After the photosynthetic gas exchange reached a steady state, the data were automatically recorded by the CIRAS-3 every 3 s. The light intensity on the surface of leaves was controlled by an automatic control device on the CIRAS-3 according to one of two protocols used in this study. The first one was as follows: 180 s of HL (1600 μmol m^− 2^ s^− 1^), 60 s of LL (100 μmol m^− 2^ s^− 1^), 300 s of HL, 120 s of LL, 300 s of HL, 300 s of LL, 1200 s of HL, 600 s of LL, and then 1800 s of HL. The second protocol was as follows: 240 s of HL followed by LL and HL alternating every 120 s. In this study, the area below the time course curve of the gas exchange represents the integrated gas exchange. The induction state (IS%) value of Pn was calculated as IS% = (Pn_30s_ + R_d_) / (Pn_steady_ + R_d_), where Pn_30s_ is the Pn after 30 s of HL following an interval of LL, Pn_steady_ is the Pn under steady-state HL conditions, and R_d_ is the respiratory rate in darkness.

### Immunoblot analysis

After steady-state gas exchange measurements, the leaves were cut, weighed and immediately stored in liquid nitrogen. The soluble proteins were extracted with a buffer consisting of 50 mM HEPES-KOH (pH 7.8), 10 mM NaCl, and 2 mM MgCl_2_ from 1.75 cm^− 2^ of leaf tissue. Ten microliters of soluble proteins were mixed with loading buffer and degenerated at 99 °C for 10 min. The soluble proteins were then loaded onto and separated on a 10% (w/w) SDS-PAGE gel. Proteins from the gel were subsequently blotted onto nitrocellulose using standard methods. Immunodetection was carried out via specific primary antibodies and horseradish peroxidase-conjugated anti-rabbit secondary antibodies (Solarbio, China). A Thermo Scientific SuperSignal West Pico substrate was applied for the detection of the immunoreaction. The chemiluminescence was recorded on blots via a Tanon 5500 cooled charge-coupled device camera (Tanon, China). The primary antibodies against fructose-1,6-bisphosphatase (FBPase), sedoheptulose-1,7-bisphosphatase (SBPase) and the Rubisco large subunit (RbcL) were purchased from Phyto AB (USA) or Agrisera (Sweden).

### Determination of enzyme activity

The enzyme activity and gas exchange were analysed in different plants. Before illumination, the leaf area was measured by an LI-3000C Area Meter (Li-Cor, USA). After illumination, the leaves were isolated from the plants and quickly frozen in liquid nitrogen. The Rubisco activity was determined in accordance with the methods of Yang et al. and Cheng et al. [66, 67]. The frozen leaf discs were ground to a fine powder in liquid nitrogen using a mortar and pestle in extraction buffer containing 100 mM tricine (pH 8.0), 5 mM MgCl_2_, 0.1 mM EDTA, 5 mM dithiothreitol, 1% (w/v) polyvinylpyrrolidone, 1% (w/v) casein and 0.05% (v/v) Triton X-100. The initial Rubisco activity was measured in reaction medium containing 5 mM HEPES-NaOH (pH 8.0), 1 mM NaHCO_3_, 2 mM MgCl_2_, 0.25 mM dithiothreitol, 0.1 mM EDTA, 1 U of glyceraldehyde-3-phosphate dehydrogenase, 0.5 mM ATP, 0.015 mM NADH_2_, 0.5 mM phosphocreatine, and 0.06 mM ribulose-1,5-bisphosphate. The change in absorbance at 340 nm was monitored for 90 s. The SBPase activity was determined in accordance with the methods of Harrison et al. and Simkin et al. [68, 69]. The extraction buffer containing 50 mM HEPES (pH 8.2), 5 mM MgCl_2_, 1 mM EDTA, 1 mM EGTA, 10% glycerol, 0.1% Triton X-100, 2 mM benzamidine, 2 mM amino caproic acid, 0.5 mM phenylmethanesulfonyl fluoride (PMSF), and 10 mM dithiothreitol (DTT).

reaction medium containing 50 mM Tris (pH 8.2), 15 mM MgCl_2_, 1.5 mM EDTA, 10 mM DTT, and 2 mM sedoheptulose-1,7-bisphosphate and extracts, the analysing before after 5 min incubation at 25 °C. The reaction was stopped by the addition of perchloric acid solution and then centrifuged at 4 °C. The samples and standards were mixed with Biomol Green (Affiniti Research Products, Exeter, UK) and then incubated for 30 min at room temperature before the absorbance at 620 nm was measured. The FBPase activity was determined according to the method of Sassenrath-Cole and Pearcy [45]. The extraction buffer containing 100 mM tricine (pH 8.1), 10 mM MgCl_2_, 1 mM EDTA, 15 mM mercaptoethanol, and 1 mM fructose-1,6-bisphosphate. The FBPase activity was determined at 25 °C by measuring the increase in absorbance at 340 nm in 1 mL of assay buffer containing 100 mM tricine (pH 8.1), 20 mM MgCl_2_, 1 mM EDTA, 0.3 mM NADP^+^, 0.6 mM fructose-1,6-bisphosphate, 0.6 units of glucose-6-P-dehydrogenase, 1.2 units of phosphoglucoisomerase and 0.1 mL of leaf extract. Some cytosolic FBPase activity will be detected despite using assay conditions to favour plastid FBPase. However, it was reported that the activity of cytosolic FBPase is only less than 1.2% of the activity of plastid FBPase in *Arabidopsis* leaves under light [70]. Therefore, the contribution of cytosolic FBPase to the FBPase activity was neglected. The activity of FBPase detected by the above assay conditions was considered plastid FBPase.

### Statistical analysis

The measurements were performed in 3–6 plants. T-tests were used to analyse differences between the treatments using SPSS 11.

## Supplementary information

**Additional file 1: Figure S1.** The stomatal conductance (Gs), transpiration rate (E) and the substomatal CO_2_ concentration (Ci) under changing light conditions.

**Additional file 2: Figure S2.** The PSII photoinhibition caused by the mensuration of dynamic photosynthetic gas exchange.

**Additional file 3: Figure S3.** The effect of osmotic potential on photosynthetic gas exchange.

**Additional file 4: Figure S4.** The stomatal conductance (Gs), transpiration rate (E) and substomatal CO_2_ concentration (Ci) under fluctuating light conditions.

**Additional file 5: Figure S5.** The original, full-length gel and blot of Rubisco, SBPase and FBPase.

**Additional file 6.** raw data of Fig. 1-5 and Fig. S1-S4.

## Data Availability

All data generated or analysed during this study are included in this published article [and its supplementary information files].

## Abbreviations

Ci: Substomatal CO_2_ concentration
Cr: CO_2_ partial pressure
E: Transpiration rate
FBPase: Fructose-1,6-bisphosphatase
HL: High light
HN: High nitrogen
IS%: Induction state of Pn
J_max_: Maximum rates of RuBP-regeneration
LL: Low light
LN: Low nitrogen
PEG: Polyethyleneglycol
PFD: Photon flux density
Pn: Photosynthetic rate
Pn_max_: Maximum Pn under the HL period
PQY: Photosynthetic quantum yield
RbcL: RUBISCO large subunit
RCA: Rubisco activase
Rd: Mitochondrial respiration rates in the light
Rubisco: Ribulose-1,5-bisphosphate carboxylase/oxygenase
SBPase: Sedoheptulose-1,7- bisphosphatase
SLA: Specific leaf area
V_cmax_: Maximum rates of RuBP-carboxylation
