# Supplementary material for: Dynamic light caused less photosynthetic suppression, rather than more, under nitrogen deficit conditions than under sufficient nitrogen supply conditions in soybean
Source: BMC Plant Biol. 2020 Jul 17;20:339. doi: 10.1186/s12870-020-02516-y (PMC7368695; doi:10.1186/s12870-020-02516-y)
Supplement: Supplementary file 2 [file 12870_2020_2516_MOESM2_ESM.docx]

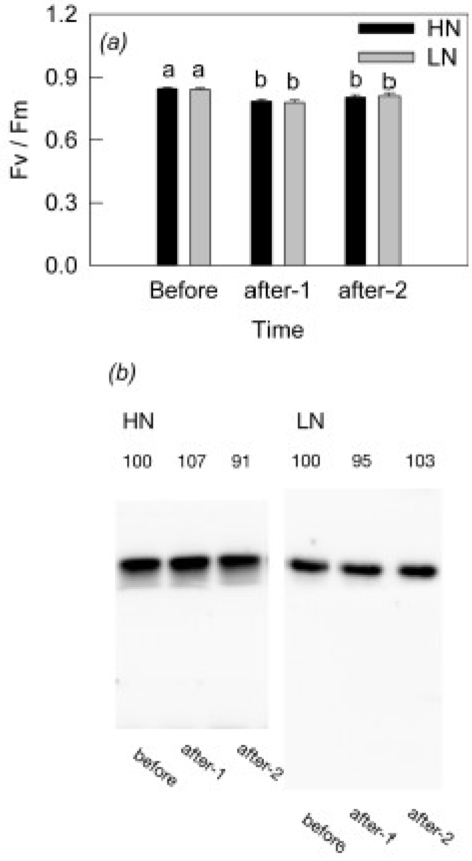

Additional file 2: Figure S2: The PSII photoinhibition caused by the mensuration of dynamic photosynthetic gas exchange.

The maximum quantum yield of PSII (Fv/Fm; *a*) and the amounts of D1 protein (*b*) in leaves before and after the dynamic photosynthetic gas exchange as showed in Fig. 2 (after-1) and Fig. 3 (after-2). The Fv/Fm measured by Handy-PEA (Hansatech, UK) from dark-adapted leaves. Means ± SD, n = 6. Different letters indicate significant differences at $P < 0.05$ between different treatments.
