# Supplementary material for: Dynamic light caused less photosynthetic suppression, rather than more, under nitrogen deficit conditions than under sufficient nitrogen supply conditions in soybean
Source: BMC Plant Biol. 2020 Jul 17;20:339. doi: 10.1186/s12870-020-02516-y (PMC7368695; doi:10.1186/s12870-020-02516-y)
Supplement: Supplementary file 3 [file 12870_2020_2516_MOESM3_ESM.docx]

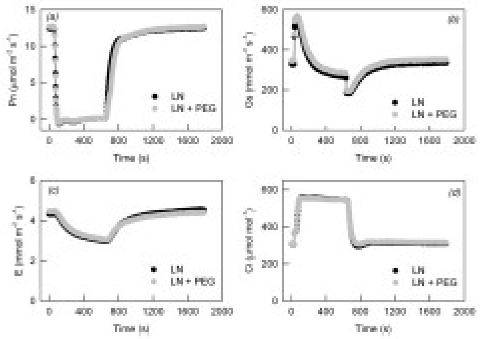

Additional file 3: Figure S3: The effect of osmotic potential on photosynthetic gas exchange. The time course of net photosynthetic rate (Pn; a), stomatal conductance (Gs; b), transpiration rate (E; c) and the substomatal CO_2 concentration (Ci; d) under changing light in leaves of low nitrogen (LN) supply plants before and 24 h after moving to lower osmotic potential nutrient solution (LN+PEG). The osmotic potential in LN+PEG nutrient solution was similar with it in HN nutrient solution.
