# Supplementary material for: Dynamic light caused less photosynthetic suppression, rather than more, under nitrogen deficit conditions than under sufficient nitrogen supply conditions in soybean
Source: BMC Plant Biol. 2020 Jul 17;20:339. doi: 10.1186/s12870-020-02516-y (PMC7368695; doi:10.1186/s12870-020-02516-y)
Supplement: Supplementary file 4 [file 12870_2020_2516_MOESM4_ESM.docx]

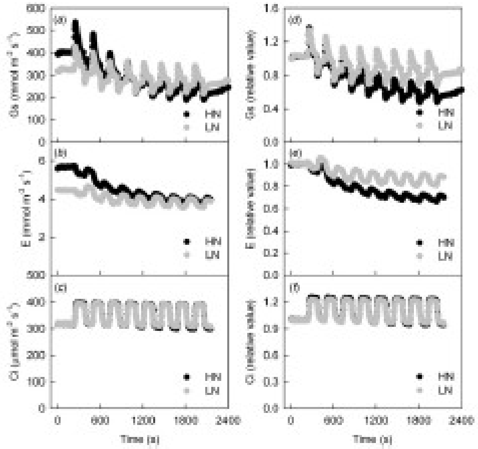

Additional file 4: Figure S4: The stomatal conductance (Gs; a, d), transpiration rate (E; b, e) and the substomatal CO_2 concentration (Ci; c, f) under fluctuating light conditions.

The time course of E and Ci under changing light in leaves of high (HN) and low nitrogen (LN) supply plants. The leaves were adapted under high light (1600 μmol m^{-2} s^{-1}) for 20–40 min until Pn stabilized, and then the leaves were exposed to fluctuating light that the light intensity was switched between high (1600 μmol m^{-2} s^{-1}) and low (100 μmol m^{-2} s^{-1}) every 120 s. The original Gs, E and Ci were shown in plot (a-c). In plot (d-f), the Gs, E and Ci under steady high light were taken as 100%, and the Gs, E and Ci under changing light condition were calculated as percentages of the steady Gs, E and Ci under high light.
