# Supplementary material for: Dynamic light caused less photosynthetic suppression, rather than more, under nitrogen deficit conditions than under sufficient nitrogen supply conditions in soybean
Source: BMC Plant Biol. 2020 Jul 17;20:339. doi: 10.1186/s12870-020-02516-y (PMC7368695; doi:10.1186/s12870-020-02516-y)
Supplement: Supplementary file 5 [file 12870_2020_2516_MOESM5_ESM.docx]

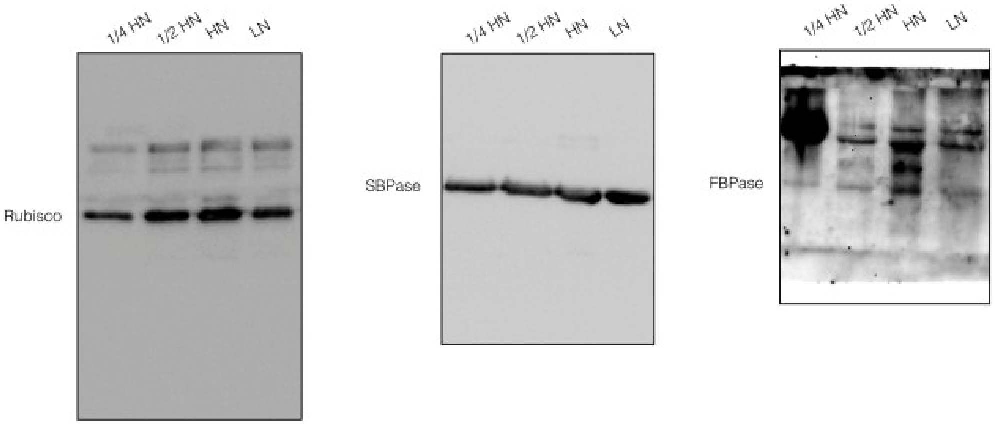

Additional file 5: Figure S5: The original, full-length gel and blot of Rubisco, SBPase and FBPase.

The amounts of Rubisco, SBPase and FBPase (e) in the leaves of high nitrogen (HN)- and low nitrogen (LN)-supplied plants. The 1/2 and 1/4 indicate the quantity of protein sample loaded.
